# Role of Dynesys as Pedicle-Based Nonfusion Stabilization for Degenerative Disc Disorders

**DOI:** 10.1155/2012/218385

**Published:** 2012-12-26

**Authors:** Neel Anand, Eli M. Baron

**Affiliations:** ^1^Minimally Invasive Spine Surgery, Department of Surgery, Cedars Sinai Spine Center, 444 S. San Vicente Boulevard, Suite 800, Los Angeles, CA 90048, USA; ^2^Department of Neurosurgery, Cedars Sinai Spine Center, 444 S. San Vicente Boulevard, Suite 800, Los Angeles, CA 90048, USA

## Abstract

Posterior nonfusion pedicle-screw-based stabilization remains a controversial area of spine surgery. To date, the Dynesys system remains the most widely implanted posterior nonfusion pedicle screw system. We review the history of Dynesys and discuss clinical outcome studies and biomechanical evaluations regarding the Dynesys system. Indications for surgery and controversies are discussed. Recommendations are made regarding technical implantation.

## 1. Introduction

Posterior nonfusion pedicle-screw-based stabilization is a controversial area of spine surgery. In the last 15–20 years, numerous devices have appeared on the market only to fall out of favor with clinical trials. Various proposed indications exist ranging from discogenic pain to fusion alternatives in the case of possible instability. Additionally, these devices have been used in the setting of artificial discs, in a hybrid construct adjacent to a fusion, and even in the setting of degenerative scoliosis. Out of all of the devices available, the largest experience is with Dynesys. We review the history and literature regarding Dynesys. We also detail our experience with Dynesys in detail and discuss lessons learned in terms of the treatment of degenerative disc disease with these technologies.

## 2. Brief History

The first commonly used posterior pedicle-screw-based nonfusion system was the Graf ligament. This was followed by the use of the Dynesys System. It has been postulated that pedicle-screw-based systems function as a tension band resulting in offloading of the disc possibly resulting in functional improvement [1–3].

Graf ligamentoplasty, introduced in the 1990s, was the first widely used pedicle-screw-based nonfusion stabilization procedure. In this procedure, braided polyester ligaments in the form of a loop were applied around pedicle screws under tension to lock an individual segment in extension [4]. In theory, this device shifted the load from the anterior part of the disc to the posterior annulus [5]. By offloading the painful anterior portion of the disc, this device theoretically may be useful in the treatment of back pain [4, 6].

Grevitt et al. [5] reviewed the outcome of 50 patients undergoing Graf ligamentoplasty. This was done primarily for degenerative disc disease. At an average followup of 24 months, they reported Oswestry Disability Indices (ODIs) scores improving from an average of 59% to 31%. Similar results were found by Gardner and Pande where they reported excellent results in 62% of patients with an average followup of 7.84 years [7]. Mean ODIs improved from 59% ± 10% preoperatively to 37.7% ± 14% after seven years. Brechbühler et al. concluded that good results were seen in patients with a combination of minor disc degeneration and mild loss of intervertebral height, fixed back musculature and facet arthritis [1].

Hadlow et al. compared the results of posterolateral fusion to Graf ligamentoplasty in a retrospective series of 83% of patients [8]. They noted a higher rate of revision with Graf ligamentoplasty of two years with better outcomes in patients managed by posterolateral fusion. While this was significant at one year, at two years the differences were no longer statistically significant. Nevertheless, the authors questioned the use of Graf ligamentoplasty.

Similarly, Rigby et al. noted poor long-term results of Graf ligamentoplasty [9]. They noted in the retrospective series of 51 patients, a 21.5% complication rate with seven patients needing to go into spinal fusion procedures. As a result, this procedure was not recommended.

The Graf ligamentoplasty had several theoretical drawbacks [6]. The device may result in increased lateral recess narrowing with hypothetical nerve root compression, and foraminal narrowing especially in the presence of preexisting stenosis [6]. Additionally, the device is applied with compression across the pedicle screws, and as a result of the compression flexion is restricted and in theory loads are increased across the posterior annulus. Increased loading of the posterior annulus may be associated with increased discogenic back pain [4, 6].

As a result of the issues with the Graf ligamentoplasty, especially with regards to compression of the posterior annulus, the Dynesys was developed. Dynesys (dynamic neutralization system of the spine) consists of pedicle screws, which are made of a titanium alloy (Protasul-100) and polyethylene terephthalate (Sulene-PET) cord and polycarbonate urethane spacers [3]. This was designed to reduce the high revision rate seen with the Graf ligamentoplasty. The polyester bands used with the Graf system were eliminated and replaced with a cord spacer between screw heads. As the spacers are not elastic, flexion results in compression of the disc in the axis of flexion of the posterior ligament. When a patient extends with Dynesys, however, the anterior annulus opens, without compression of the posterior annulus, and the system theoretically unloads the disc space. This is especially true when the patient is maintaining a lordosis and when the spacers become weight bearing [6]. This is specifically because the device limits extension as opposed to the Graf ligamentoplasty.

Schmoelz et al. demonstrated that the Dynesys system offloads the disc in extension as conveyed in biomechanical testing [10]. Thus, the problem seen with the Graf ligamentoplasty resulting in posterior annulus related compression is avoided. MRI has also shown a reduction in overall motion of the instrumented segment with Dynesys [11]. Beastall et al. confirmed no reduction in posterior disc height, while a small decrease was noted in anterior disc height [11]; they also noted no significant differences in the patient's segment motion when comparing adjacent levels to preop.

## 3. Series with Positive Results with Dynesys Implantation

Stoll et al. reported very good results in 83 patients undergoing Dynesys in a multicenter trial [12]. The implants were placed for a variety of degenerative disorders including disc herniation revision surgery and spinal stenosis. The authors noted no screw breakage, but they reported 9 failures out of 83 cases, 4 required early revision, 7 screw loosening, and 7 cases with adjacent segment degeneration. They postulated that the lack of breakage might be due to the elasticity of the spacers-cord combination, which may cause a cyclic peak load on the implant to be lower than in rigid constructs. They also theorized that offloading of the discs might result in reduced disc degeneration. Putzier et al. corroborated this comparing the results of 49 patients undergoing microdiscectomy alone versus 35 undergoing microdiscectomy with addition of Dynesys stabilization [2]. They noted that patients with Dynesys had no further degenerative disc changes in height configuration or morphology. They hypothesized that this was due to neutralization of disc pressures and offloading of the facet joints. Nevertheless, followup for these theories was quite short, and it has been suggested that longer followup is necessary before concluding that Dynesys is actually effective in reducing disc degeneration. Also, the authors noted that patients with marked degeneration or spinal deformity are inappropriate candidates for Dynesys implantation [2].

Bordes-Monmeneu et al. reported good outcomes with Dynesys [13]. They reported two-year outcome in 94 patients undergoing Dynesys implantation for disc herniation, degenerative disc disease, and lumbar stenosis. Overall, they reported a good outcome with ODI decreasing from 56.8% to 21.4%. They concluded that Dynesys may be useful incorporating the functionality concept as opposed to restricting movement.

Lee et al. also noted good outcomes with Dynesys [14]. They reported on 20 consecutive patients who underwent decompression and dynamic stabilization with the Dynesys system. This was done for various degenerative disorders including spinal stenosis with degenerative spondylolisthesis, degenerative spinal stenosis, adjacent segment disease after fusion, and spinal stenosis with degenerative scoliosis. They noted in a mean followup period of 27.25 months that the mean VAS decreased from 8.5 to 2.2. The ODIs improved from 79.58% to 22.17%. They also noted no cases of implant failure. They concluded that Dynesys could preserve the motion of stabilized segments and provide clinical improvement in patients with degenerative spinal stenosis with instability. They concluded that Dynesys might be a viable alternative to spinal fusion.

Long-term good outcomes with Dynesys were reported by Schaeren et al. [15]. They reported a minimum four-year followup of spinal stenosis with degenerative spondylolisthesis treated with Dynesys and decompression. Twenty-six patients were followed with a mean age of 71, who underwent surgical treatment. They noted at a minimum followup of four years that VAS scores improved significantly and that radiologically their spondylolisthesis did not progress and motion segments remained stable. They did note 1 patient to have screw breakage with low back pain. At a 4-year followup, 47% of patients also showed some degeneration of adjacent segments. Overall, patient satisfaction remained high; 95% of patients would undergo the procedure again. They concluded that this procedure would be useful for spinal stenosis and degenerative spondylolisthesis. They highlighted the lack of bone grafting need with Dynesys and thus lack of graft harvest morbidity. They did note, however, that degenerative disc disease is progressive and degeneration of adjacent segments continues to be a problem.

Good results were also reported by Hu et al. on 32 patients who underwent posterior laminectomy and Dynesys implantation for spinal stenosis with spondylolisthesis or lumbar disc protrusion [16]. All patients were followed for six to 23 months with a mean followup of 16.4 months. They noted ODIs to improve from preoperative 69% to postoperative 28%. They noted VAS scores to also be significantly improved. They noted no loosening of screws, cords, or polyester spacers. They concluded that Dynesys, combined with decompression, could achieve satisfactory and short-term clinical results in lumbar degenerative disease.

Yu et al. compared 35 patients who received Dynesys implantation at three segments ranging from L1 to S1 with 25 patients with the same indications who underwent three-level posterior lumbar interbody fusion (PLIF) [17]. All patients had a three-year followup. They noted the Dynesys patients to have a higher preservation of motion at operative levels as well as total range of motion. They also noted that a decrease of anterior disc height was seen in the Dynesys group, while an increase was seen in the posterior lumbar interbody fusion group. Overall, however, they noted the Dynesys group showing a greater improvement in Oswestry Disability Index and Visual Analog Scale back pain at three years postoperatively. They concluded that Dynesys was an acceptable alternative to PLIF for the treatment of multisegment lumbar disease [17].

Kim et al. compared outcomes of single-versus multilevel dynamic stabilization using Dynesys [18]. They noted 21 patients were evaluated, on average at 31 months postop. Single-level Dynesys versus multilevel Dynesys were groups were compared. They noted that the disc height was preserved in both groups. However, they reported retrolisthesis in adjacent segments above in six patients within the group that had a multi-level Dynesys implantation. The authors concluded that dynamic stabilization is a good alternative treatment option for degenerative spinal disease. However, dynamic stabilization preserves only limited motion and may cause stress on the adjacent level above. They caution to observe adjacent segment disease closely, especially in the cases of multi-level instrumentation.

## 4. Series with Negative Results with Dynesys Implantation

Würgler-Hauri reported on 37 consecutive patients with acquired lumbar stenosis and instability that underwent lumbar microsurgical decompression and implantation of Dynesys [19]. They followed the patients at three months and at 12 months. They noted a decrease in pain from 59.2% to 27.3% after microsurgical decompression. They also noted, however, a high complication rate including four broken and two misplaced pedicle screws within a total of 224 screws implanted. They also noted two loosened systems and one cerebrospinal fistula. At one year, a total of 7 patients (19%) required surgical revision. They concluded that “the reported biomechanical principles of Dynesys do not reflect the advantages and outcomes compared with none or other stabilization systems after microsurgical and radicular decompression reported in the literature.”

Grob et al. reported the results of 31 patients who underwent Dynesys implantation [20]. While they reported a 67% improvement rate, they noted a late reoperation rate as high as 19%. Grob's series assessed Dynesys implanted for a variety of indications including lumbar stenosis, spondylosis, disc degeneration, failed back surgery, degenerative listhesis, and extradural tumor. Given the small series and the very heterogeneous group if indicated, it was difficult to draw any firm conclusions. Nevertheless, Grob's feeling was that the device was not superior in any way to posterolateral fusion.

## 5. United States Food and Drug Administration (FDA) Study

Welch et al. optimistically reported preliminary results from the Food and Drug Administration investigational device exemption clinical trial of Dynesys [21]. They noted significant improvement at one year both in leg and back pain (ODI scores improved from 80.3% to 25.5% and from 55.6% to 26.3%, resp.). The authors noted that Dynesys might be preferable to fusion for surgical treatment of degenerative spondylolisthesis and stenosis because it decreases back and leg pain, while avoiding the greater tissue destruction and morbidity of donor site problems encountered in fusion. They also noted that this might be related to Dynesys preserving the disc, unloading the facet joints, and permitting more normal motion.

Nevertheless, when the FDA reported their Executive Summary on the Dynesys system in November 2009, their conclusions were not favorable [22]. Three hundred sixty-seven patients were randomized to Dynesys or fusion using posterior pedicle screws and autogenous bone grafting. Inclusion criteria included degenerative spondylolisthesis or retrolisthesis and/or spinal stenosis, lumbar radiculopathy, leg pain greater than back pain, among others. Patients were prospectively evaluation preoperative, intraoperatively and postoperatively at 3 weeks, 3, 6, 12, and 24 months. Complications and adverse events were noted. Dynesys outcomes were noted to be superior to fusion success rate for VAS leg pain (87% versus 73%), ODI success (76% versus 70%), and neurologic success (92% versus 84%) at 24 months postop. The FDA noted that the overall clinical success results of Dynesys were noninferior to the fusion (52% versus 40%). The FDA advisory committee concluded, however, that the data here was unclear and that changes should have been made to the clinical trial. They recommended against the wider use of the system.

## 6. Biomechanics and Adjacent Segment Degeneration with Dynesys Implantation

In a nonhuman primate model, Cunningham et al. studied 14 baboons where Dynesys was implanted [23]. Eight animals underwent spinal surgery to implant Dynesys spanning two levels. Six animals were sacrificed acutely and their spines were biomechanically tested in the intact condition with instrumentation implanted. They noted that a flexion/extension motion for the acute group of instrumented spines was 27% of intact condition. After six months with instrumentation in situ, flexion/extension was 56% of the intact condition. After 12 months with instrumentation in situ, flexion/extension was 70% of the intact condition. With the instrumentation explanted, flexion/extension at six and 12 months was not different from the intact condition (*P* > 0.05). They noted similar results for lateral bending. They noted no significant differences in axial rotation between any of the groups at any point. The facet joints at the operative and adjacent levels exhibited normal articular cartilage at both six and 12 months postoperative time points. They did report, however, that after 12 months 25% rate of screw loosening was noted.

Delank et al. compared in a biomechanical study rigid pedicle screw fixation versus Dynesys [24]. They noted an almost equal reduction in flexion and extension and right and left bending for both the rigid and dynamic systems. At the adjacent segments, they noted a slightly higher mobility for rigid stabilization than for dynamic stabilization. They noted that in rigid stabilization that the first cranial segment has compensatory flexion/extension movement, while in the cases of dynamic stabilization the compensation is distributed among the first and second crania and by 20% in the caudal adjacent segment. They concluded that rigid and dynamic stabilization do not result in the significant changes in range of motion of the instrumented segment. However, the distribution of compensatory adjacent segment movement is different.

A radiographic study of motion and alignment of patients after Dynesys implantation was conducted by Cakir et al. [25]. Twenty-six patients undergoing either decompression and fusion or decompression and Dynesys. They were followed radiographically with flexion/extension radiographs. Based on image analysis, they concluded that there was significant reduction in the global range of motion of the lumbar spine and the segmental range of motion at the index level in the fusion groups, where adjacent level range of motion did not change significantly. In the Dynesys group, they noted no significant changes in global lumbar spinal movement and segmental range of motion. They concluded that neither monosegmental instrumented fusion nor Dynesys altered the range of motion of the cranial or caudal adjacent segments. Consequently, Dynesys may have no effect with regard to adjacent segment mobility when compared to monosegmental fusion.

Strube et al. reported on biomechanical results in a cadaveric model of Dynesys implantation versus rigid instrumentation implantation [26]. The authors noted that both dynamic and rigid fixation of L4-L5 adjacent to rigid fixation of L5-S1 led to an increase of the mean range of motion of L3-L4 in extension, flexion, lateral bending, and left axillary rotation and at L2-L3 in all major planes of motion. Compared to single-level fixation with segmental instrumentation, both dynamic and rigid ones led to a further increase in the mean range of motion for flexion and right lateral bending, whereas axial rotation and extension seemed not to be significantly affected by the additional fixation at L4-L5 superior to rigid fixation. They concluded that hypermobility of the adjacent levels increased with the number of fixated levels. They also noted effects of Dynesys implant to be very close to those of rigid fixation since there were no differences at L3-L4 between any of the dynamic or rigid fixation when done above a fusion. They noted a slight difference in lateral bending when dynamic fixation was used versus rigid fixation. They concluded, however, that dynamic instrumentation cannot be recommended if prevention of hypermobility in the adjacent level is a main target.

Beastall et al. reported on 24 patients with predominantly low back pain with or without leg pain that were treated with Dynesys [11]. All patients underwent positional MRI before surgery and nine months after surgery. Measures were made to assess the differences at the operated level, the adjacent, and whole lumbar spine. They noted that statistically significant reduction in flexion and extension range of movement both of the whole lumbar spine by 13.37° and at the instrumented segment by 4.8° following the surgery. There was an insignificant reduction in the range of movement of the level of above the instrumentation. Mean anterior disc height at the instrumented level reduced by 0.7 mm following insertion of the Dynesys. Mean posterior disc height reduced by 0.3 mm. In neutral posture, they noted the Dynesys to have no significant impact on lordosis or inclination of the operated or adjacent levels. They noted the Dynesys to appear to restrict extension more than flexion with respect to neutral posture. They concluded that Dynesys allowed movement at the instrumented level, albeit reduced, with no significant increase in mobility at the adjacent segments, but with reduction of the anterior disc height without significant increase in posterior disc height.

Interestingly, Beastall et al.'s in vivo findings were opposite to the earlier cadaver model findings of Schmoelz et al., where Dynesys was found to stabilize the spine least in extension, where motion resembled the intact spine [27]. In their cadaveric study, Schmoelz et al. noted Dynesys to stabilize the spine, but they noted it to be more flexible than a rigid external fixator. They noted the greatest difference between the two devices in extension. They also noted that adjacent segment motion was not affected by either form of fixation. In a later study, with a similar model, Schmoelz et al. studied the effect of Dynesys versus rigid fixation on disc loading [10]. They noted both Dynesys and the rigid fixator to significantly reduce intradiscal pressure in extension and in lateral bending compared to the intact state. There was no significant change in intradiscal pressure with flexion with either form of fixation. They also noted no changes in intradiscal pressure in the adjacent discs for either the Dynesys or the internal fixator. Liu et al. studied Dynesys implanted in a three-dimensional nonlinear finite-element model of the L1-L5 lumbar spine [28]. Range of motions and stress was studied. Under flexion, extension, and lateral bending, Dynesys provided significant stability at the surgical level but increased range of motion at the adjacent level. Under flexion and lateral bending, the Dynesys alleviated annular stress at the surgical level but was noted to increase annular stress at the adjacent level. Under extension, Dynesys decreased facet loading at the surgical level but increased facet loading at the adjacent level. The authors concluded that Dynesys was able to restore stability and alleviate loading on disc and facet at the instrumented level, but they noted at adjacent levels greater range of motion, annular stress, and facet loading were found. In addition, they noted that profile of screw placement caused only a minor influence on the range of motion, annular stress, and facet loading, but screw stress was notably increased.

## 7. Hybrid Dynamic Stabilization and Fusion System

Maserati et al. described the use of a hybrid dynamic stabilization and fusion system where Dynesys was used in conjunction with Optima pedicle screws in the setting of Dynesys being used for dynamic stabilization above fusion, with transforaminal lumbar interbody fusion being performed at the caudal segment [29]. They reported on 24 consecutive patients. Indication for implantation of the hybrid system was unclearly reported. They stated that “patients with degenerative lumbar disc disease were chosen to undergo the procedure if they were candidates for fusion and had symptomatic adjacent level pathology in which dynamic stabilization was thought to be more appropriate than arthrodesis.” They noted, at followup of eight months, VAS score to be improved. Additionally, they noted five perioperative complications including two dural tears and one case of medially placed pedicle screws. They also noted 3% of their patients to have failure requiring extension of their fusion. They noted that 1 patient developed symptomatic degeneration of the dynamic stabilized segment and 2 patients had symptomatic degeneration above the dynamically stabilized segment.

In another study on hybrid dynamic stabilization, Putzier et al. reported on the use of Dynesys adjacent to a single-level fusion [30]. In a study designed to compare dynamic fixation of a clinically asymptomatic adjacent segment disease with circumferential lumbar fusion alone, 60 patients with symptomatic degeneration of L5-S1, L4-L5, and asymptomatic adjacent segment degeneration were divided into two groups. Thirty patients were treated with circumferential single-level fusion and 30 were treated with a dynamic fixation and transition above the level of fusion. Patients were assessed postoperatively at 12 months and a mean followup of 76.4 months. Radiologic parameters in addition to clinical parameters were followed. At final followup, two nonfusions were observed in both groups. Six single-level fusion patients and one dynamically fixed transition patient presented with a progression of adjacent segment disease. In 2 of the dynamic fixation transition patients, however, symptomatic progressive adjacent segment degeneration occurred in the segment superior to the dynamic fixation. In 1 of the patients with Dynesys, the fusion of the dynamically fixated segment was observed. Four of the dynamically fixated transition patients presented with radiologic implant failures. The authors recommended against dynamically fixating adjacent segments in patients with clinically asymptomatic adjacent segment degeneration. The reduced numbers of progression of adjacent segment degeneration seen with dynamic fixation were accompanied by a higher number of implant failures and a shift of adjacent segment degeneration to a shift above the superior segment.

## 8. Controversies and Controversial Indications

Vaga et al. reported molecular MRI imaging for an evaluation of the effect of Dynesys on lumbar intravertebral discs [31]. Ten patients with low back pain unresponsive to conservative treatment underwent implantation of Dynesys at one to three spinal levels. Subsequently, authors assessed the quantification of glycosaminoglycan (GAG) concentration within instrumented and adjacent levels by mean of delayed gadolinium enhanced MRI imaging of cartilage protocol. At six months after implantation, they noted VAS and Oswestry scores to improve. They noted GAG to increase in 61% of the instrumented levels, while 68% of the noninstrumented levels showed a decrease in GAG, mainly in the posterior disc portion. The authors concluded that dynamic stabilization of the lumbar spine is able to stop and partially reverse disc degeneration especially in seriously degenerated discs, while increasing the stress at the adjacent levels.

Their findings, however, were contradicted in a study by Kumar et al. where 32 patients who underwent Dynesys procedure had their discs assessed at 2 years postop. using MRI and the Woodend disc degeneration scoring system [32]. They noted disc degeneration to increase in both the bridged and adjacent levels when compared to preop.

Di Silvestre et al. reported outcomes of 29 elderly patients with mild degenerative scoliosis (mean Cobb angle 16.9°) who underwent Dynesys with laminectomy [33]. Mean followup was 54 months. ODI, back pain, and leg pain improved considerably. Mean Cobb improved from 16.9 to 11.1 degrees. Additionally associated spondylolisthesis was stable. They noted 4 cases of asymptomatic radiolucencies around S1 screws. They concluded that Dynesys with laminectomy provided enough stability to prevent progression of scoliosis and instability.

Another unusual indication for Dynesys is correction of disc tilt after total disc replacement. Cheng et al. reported on 3 patients presenting with tilted total disc replacement [34]. All patients underwent corrective surgery with Dynesys, where the collapsed side was expanded and the contralateral side was compressed using manipulation of the universal spacer.

Ko et al. reported on screw loosening in a Dynesys stabilization system [35]. They reviewed the charts, radiographic films, and medical records of 71 patients who underwent decompression and Dynesys dynamic stabilization for one or two levels. Mean follow-up duration was 16.6 months and 71 patients were studied. They noted an overall radiographic evidence of loosening in 19.7% of patients and 4.6% of screws. Nevertheless, they noted no adverse effect on clinical improvement. The followup, however, was relatively short.

## 9. Indications for Dynesys

**Figure 1:**
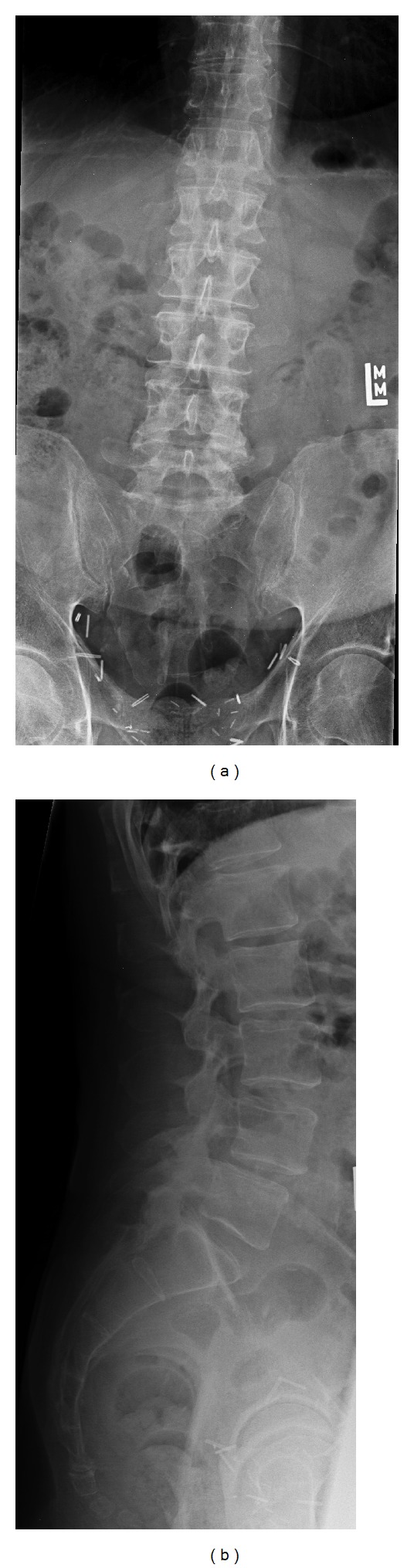
(a) and (b) Anteroposterior and lateral radiographs of a 63-year-old gentleman with severe left lower extremity pain unresponsive to conservative measures. Workup revealed him to have an L4-5 degenerative spondylolisthesis in addition to having a left-sided juxtafacet cyst compressing the descending root of L5. The patient underwent L4-5 Dynesys placement in addition to laminoforaminotomy, mesial facetectomy, and excision of the facet cyst.

**Figure 2:**
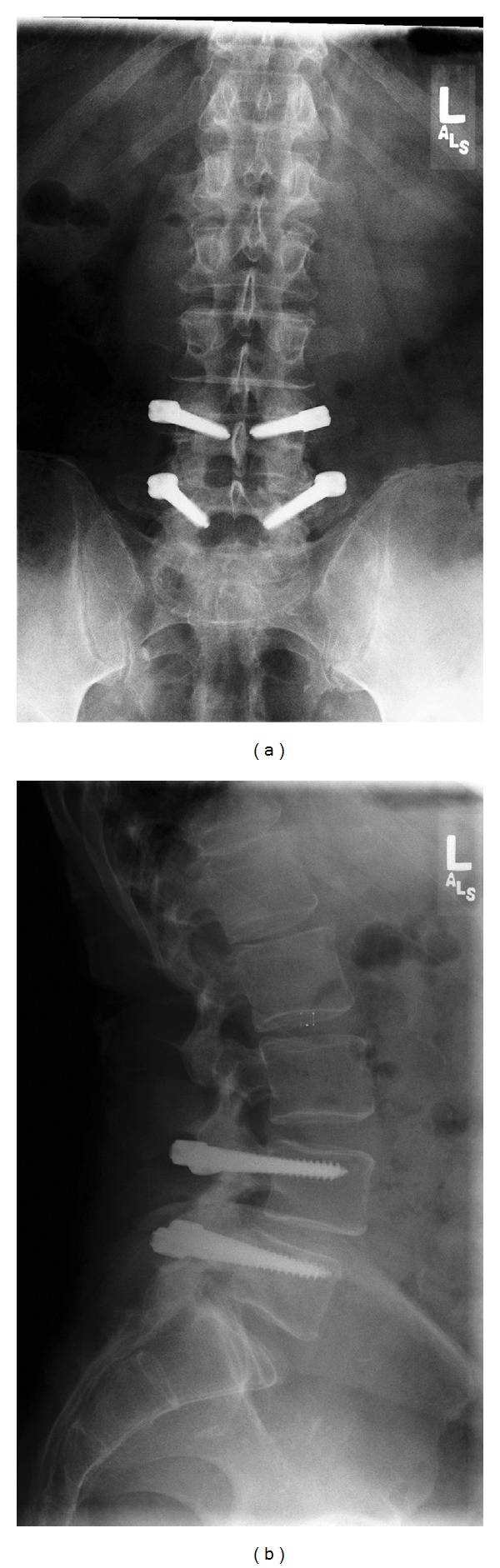
(a) and (b) Postop. anteroposterior and lateral radiographs at 1 year showing instrumentation intact and disc height comparable to preop.

**Figure 3:**
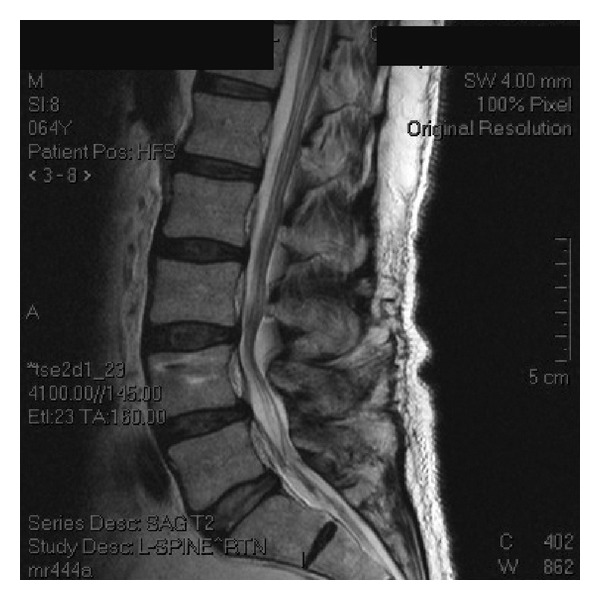
Midsagittal MRI at 2 years outdemonstrating maintenance of disc configuration at L4-5 and a well-hydrated supraadjacent segment.

**Figure 4:**
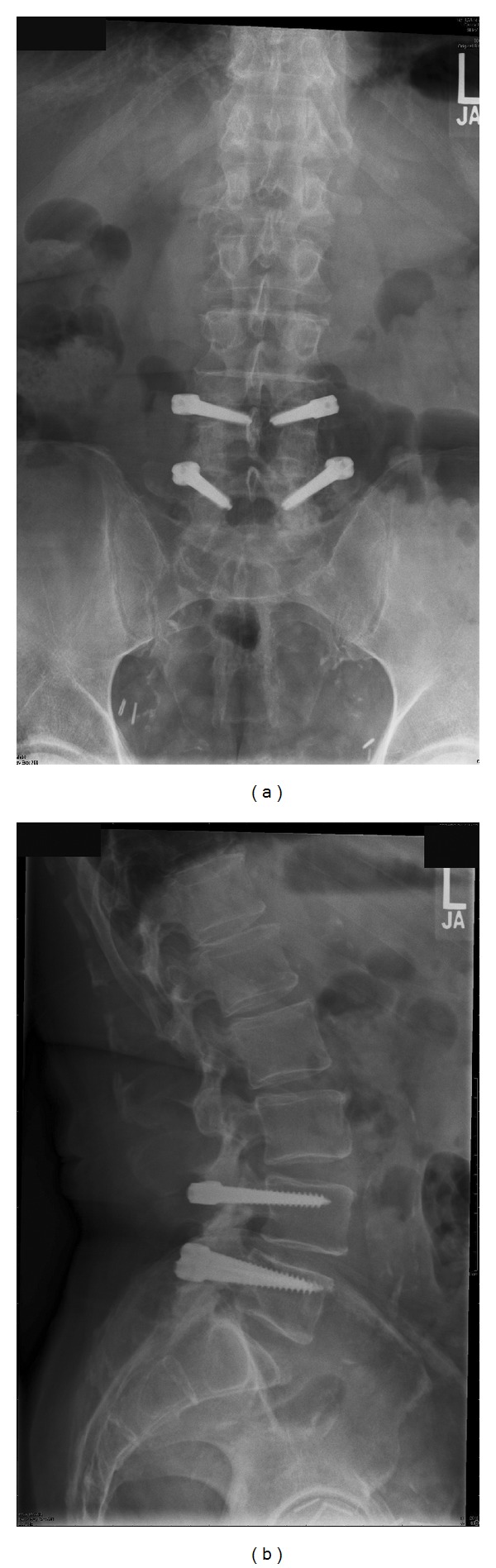
(a) and (b) Postop. anteroposterior and lateral radiographs at 7.5 years postop. showing instrumentation intact and disc height comparable to preop.

Though as indicated above, many proposed indications of dynamic stabilization have been used. In our experience, we have had best results in patients who have mild-to-moderate degeneration with mechanical loading back pain with no secondary gain factors. Our ideal patient would have mechanical loading back pain limited to one or two segment disease. The following radiologic factors would be acceptable: disc collapse less than 50% of normal, up to grade 1 degenerative spondylolisthesis, translational motion less than 3 mm on flexion/extension, not more than grade 1 or 2 facet degenerative changes, no lateral listhesis, no scoliotic tilt or segmental collapse, and bone density *T* score greater than −1.5 (Figures 1, 2, 3, and 4). Stenosis of any degree at the involved levels is not a contraindication. We have performed a microscopic laminoforaminotomy to achieve decompression, avoiding a laminectomy.

We reported outcomes on 88 patients who underwent Dynesys both using the muscle sparing paraspinal approach versus midline approach [36]. At mean followup of 18 months, both groups showed significant improvements in terms of VAS, ODI, SF-36 outcomes, and treatment intensity score (TIS, which reflects narcotic use). For the first 6 weeks postop., however, the paraspinal approach group had significantly better TIS scores. This trend continued, but was not statistically significantly at 6 months. Thus, we prefer the paraspinal approach. Additionally we reported on 35 patients undergoing Dynesys stabilization for back pain from lumbar degenerative disc disease [37]. The average age was 44 years with 22 females and 14 males. All patients had a clear history of discogenic back pain with no leg pain, further corroborated by MRI and a discogram. Average followup was 18 months. Patients did well with Dynesys where their VAS scores were reduced from 9 to 2.5. Additionally, there was significant reduction in SF-36 and ODI scores.

## 10. Technical Notes for Implantation of Dynesys

All patients are positioned prone on a Jackson table with care taken to maximize lordosis. All patients are operated through a bilateral paraspinal muscle sparing approach. Patients who need a decompression are operated on through a midline fascial approach with the skin on the symptomatic side retracted to the midline. Microscopic laminotomy or laminoforaminotomy, including decompression of the opposite side when indicated is done, with or without discectomy followed by posterior nonfusion stabilization with the facet capsule and facet joints carefully preserved. The midline structures are preserved in all cases with great care being taken to suture the lumbodorsal fascia back to the midline at the end of the procedure. No patients undergo laminectomy. Patients not needing decompression are purely instrumented with bilateral paraspinal muscles sparing approaches. In this manner, tissue damage, especially of the multifidus, is minimized. Additionally, care is taken not to violate the facet capsules or any of the muscle attachments. We feel it critical to preserve the soft tissues when nonfusion stabilization is performed.

As regards the approach, we prefer the term modified muscle sparing approach, where plane is teased apart by using a Langenbeck elevator where the fibers of the multifidus are teased medially and the longissimus laterally, demonstrating a clear cleavage plane. The transverse process for the instrumented pedicle is palpated and confirmed radiologically. We then use a narrow McCulloch blade retractor laterally and a shorter blade medially for retraction directly over the transverse process. This can be done very cleanly with minimal-to-no muscle bleeding encountered if the proper plane is identified. We have described this technique elsewhere [38]. In terms of pedicle screw insertion, care must be taken not to violate the facet joints. We typically expose the transverse process, mammillary process, and pars only without exposing the facet capsule. This, we feel, may be important in minimizing the chance of further facet degeneration in supra-adjacent segment disease. Screws are placed in a converging fashion from a far out-to-in direction with care taken not to violate the facet. Screws as long as safely possible without being bicortical are used with the screw head placed as low as possible at the junction of the superior facet and transverse process. 

Tensioning of the spacer and choosing the correct size of the spacer has been the most difficult to accurately reproduce and quantify. The proprietary tensioner available on the set gives some indication as to the amount of tension between the screws and in determining the size of the spacer, but yet this step in the procedure remains an art and difficult to quantify with any scientific accuracy. This in itself maybe partially responsible for the wide disparity in results reported amongst different series. The issue of tensioning each segment becomes even more relevant when multiple levels are instrumented and in our experience when three or more levels were instrumented the results were less than optimal as compared to one- or two-level instrumentation.

Given the benefits we have seen for the paraspinal approach, we prefer to use this approach for all patients having pedicle screw posterior nonfusion stabilization and feel once again that the approach for this technology may be an important factor determining clinical outcomes.

## 11. Conclusions

There has been considerable experience both clinically and with biomechanics with regard to the Dynesys posterior nonfusion pedicle-screw-based systems. Nevertheless, it is our belief that heterogeneous indications and heterogeneous techniques during implantation may result in less than desired outcomes. In carefully selected patients, we believe the technology to be quite useful. As evidenced by certain biomechanical and radiologic studies above, there may be a use in the future where the disc needs to be stabilized, for example, with stem cells or other restorative technologies. In the meantime, especially where early disc degeneration is present and low-grade spondylolisthesis is present, it may be useful for selected patients.
